# The Impact of COVID-19 Pandemic on Management and Outcome in Patients with Heart Failure

**DOI:** 10.3390/jcm10235577

**Published:** 2021-11-27

**Authors:** Zahi Abu Ghosh, Donna R. Zwas, Andre Keren, Gabby Elbaz-Greener, Vered Israeli, Offer Amir, Israel Gotsman

**Affiliations:** 1 Heart Institute, Hadassah Medical Center, Faculty of Medicine, Hebrew University of Jerusalem, Jerusalem 91120, Israel; zahi.abugosh@gmail.com (Z.A.G.); donnaz1818@gmail.com (D.R.Z.); kerena@mail.huji.ac.il (A.K.); Gabbyelbaz@yahoo.com (G.E.-G.); oamir@hadassah.org.il (O.A.); 2 Heart Failure Center, Jerusalem District, Clalit Health Services, Jerusalem 42849, Israel; 3 Jerusalem District Management, Clalit Health Services, Jerusalem 42849, Israel; Veredi@clalit.org.il

**Keywords:** COVID-19, SARS-CoV-2, heart failure, outcome

## Abstract

Background: The COVID-19 pandemic has adversely affected the provision of health care and disease management around the world. COVID-19 carries a high morbidity and mortality rate in elderly and people with comorbidities, including heart failure (HF). The present study addressed the clinical management and outcomes of HF patients during the pandemic. Methods: We evaluated the clinical management and survival rate of HF patients during the COVID-19 pandemic in Israel (March 2020–April 2021). Results: The cohort included 6748 patients with a diagnosis of HF during the study period. During this period, 843 HF patients (12.5%) were infected with COVID-19, and 194 died from COVID-19, a 23% mortality rate. Patients infected with COVID-19 had a higher percentage of diabetes and obesity. Predictors of mortality included age, male sex, reduced functional capacity, renal dysfunction, and absence of renin–angiotensin system inhibition. During the pandemic, there was a marked decrease in the usage of medical services in the cohort. Cardiovascular hospitalizations, all hospitalization, and emergency room visits were significantly decreased compared to the two years prior to the pandemic, particularly during the lockdowns. There was also an initial decrease in HF clinic visits. Mortality rates were very similar during the pandemic compared to previous years. There was a decline in non-COVID-19 deaths, which were replaced with deaths due to COVID-19. This may result from competing effects and reduced exposure to respiratory infections and other insults due to social distancing. Conclusions: Mortality rates in HF patients infected with COVID-19 were high. The COVID-19 pandemic resulted in the reduced usage of health services but without increased overall mortality.

## 1. Introduction

Coronavirus disease 2019 (COVID-19), which is caused by the novel coronavirus SARS-CoV-2, emerged in Wuhan, China, in December 2019 and has evolved into an international public health crisis. It was declared a pandemic by the World Health Organization on 11 March 2020 [1]. By October 2021, the SARS-CoV-2 pandemic has resulted in more than 240,000,000 cases and more than 4,950,000 deaths worldwide, including 1,320,000 cases and 8000 deaths in Israel [2].

The clinical manifestations of COVID-19 range from asymptomatic or mild respiratory symptoms to severe life threating respiratory and cardiac failure [3]. Cardiac injury, in addition to respiratory failure, is associated with a higher risk of mortality [4,5]. Cardiac manifestations may range from asymptomatic myocardial injury to cardiovascular complications, such as heart failure, acute coronary syndrome (ACS), myocarditis, pericarditis, vasculitis, cardiac arrhythmias, and cardiogenic shock [4,5,6].

Patients with concomitant cardiovascular disease represent a large proportion of patients with symptomatic COVID-19 and experience disproportionately worse outcomes and increased mortality [6,7]. New-onset or the exacerbation of heart failure (HF) are common complications in patients with COVID-19, with documented high mortality rates as high as 36% [8]. Furthermore, almost half of the patients who died from COVID-19 and who had developed HF had no previous history of either hypertension or cardiovascular disease [9].

HF patients are clearly at increased risk in the setting of the COVID-19 pandemic. There are limited data in the literature regarding the clinical management and the outcomes of HF patients during the pandemic period. The current study evaluated the clinical management and survival rate of HF patients in the Jerusalem District in Israel during the COVID-19 pandemic period and compared it to data prior to the pandemic.

## 2. Methods

Clalit Health Services is the largest health maintenance organization (HMO) in Israel. It has a central computerized database in which all members have a nearly complete digital record. The database includes demographic data and comprehensive clinical data including complete administrative data on all hospitalizations and medical visits as well as all diagnoses and laboratory data undertaken in a single centralized laboratory of the HMO. We identified and retrieved all of the members with a diagnosis of HF as coded by the database in Jerusalem, Israel electronically from the computerized database. Data were retrieved from 1 January 2018. A total of 8800 patients in this district had a diagnosis of HF and were included in the database for the analysis of medical management and overall clinical outcomes. The clinical events that were retrieved included all cardiovascular hospitalizations, emergency room visits, all hospitalizations, HF clinic visits, and mortality until 1 April 2021. HF clinic visits included actual or remote visits. A comparison of these clinical events was performed per year over the period of the study (3 years and 3 months). In order to assess the prevalence of COVID-19 infection in HF patients, predictors of infection as well as clinical outcome, a separate analysis was conducted that only included patients who were alive at the beginning of the epidemic in Israel on 1 March 2020, the date marking the first COVID-19 infections in Israel. This included all of the patients who had been previously diagnosed with heart failure and those who received a new diagnosis of heart failure during this period. A total of 6748 patients were included in this analysis and were the main cohort of the study during the pandemic study period (1 March 2020 to 1 April 2021). The determination of the type of HF, HF with reduced ejection fraction, and HF with preserved ejection fraction was based on a documented specific diagnosis and was available for 67% of the patients. The diagnosis of the remaining patients was “Heart failure, unspecified”. Natriuretic peptides are not routinely performed in Israel and were not available for analysis. All hospitalizations as well as cardiovascular hospitalizations were retrieved and analyzed; cardiovascular hospitalizations were defined as hospitalization within the cardiac or internal medicine departments, including the cardiac and internal intensive care units. Data on mortality were retrieved from the National Census Bureau. Data regarding influenza infections were obtained from the Israel Center for Disease Control respiratory viruses surveillance reports published by the Israeli Ministry of Health. The Institutional Committee for Human Studies of Clalit Health Services approved the study protocol (approval no. 0025-17-COM2).

Infection with COVID-19 was diagnosed with a standard PCR test on a swab sample from the nasopharynx. Death attributed to COVID-19 was defined as a positive diagnosis of SARS-CoV2 infection by PCR testing and subsequent death due to this infection. Biochemical analyses were performed at the HMO single centralized core laboratory with routine standardized methodologies on fresh samples of blood obtained after an overnight fast. Biochemical analyses were performed on the serum. The laboratory was authorized to perform tests according to the international quality standard ISO-9001.

SPSS version 17.0 for Windows (SPSS Inc., Chicago, IL, USA) was used for the analyses. Comparison of the clinical characteristics and medical service usage was performed using the Mann–Whitney U test or the Kruskal–Wallis nonparametric test for continuous variables and the Chi-square test for categorical variables. Follow-up time was calculated using the Kaplan–Meier estimate of potential follow-up. Kaplan–Meier curves with the log-rank test were used to compare survival according to years. Clinical predictors were transformed where appropriate. Log10 was used for logarithmic transformations with the exception of the estimated glomerular filtration rate (eGFR); for that, a square root transformation was used. Multivariate Cox proportional hazards regression analysis was used to evaluate the independent variables that determined the survival of COVID-19-infected patients. Parameters included in the multivariate Cox regression analysis incorporated age and other clinically significant parameters as well as significant laboratory parameters and drug therapy on univariable analysis. Proportionality assumptions of the Cox regression models were evaluated by log–log survival curves and with the use of Schoenfeld residuals. An evaluation of the existence of confounding or interactive effects was made between variables and their possible collinearity. A *p* value of <0.05 was considered statistically significant.

## 3. Results

### 3.1. COVID-19 Pandemic in Israel

**Figure 1:**
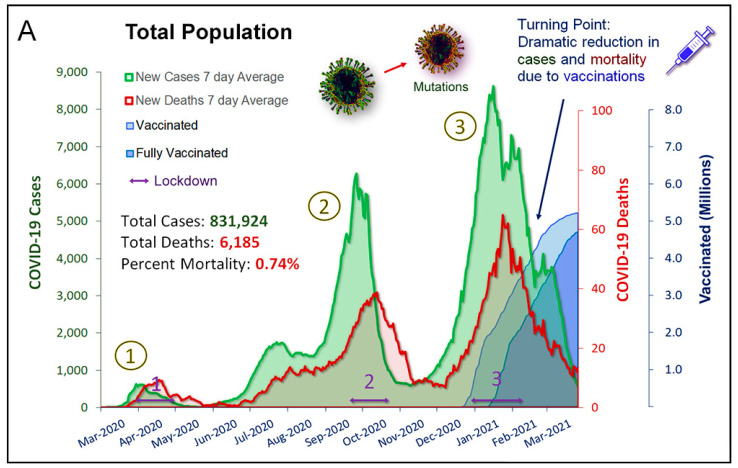

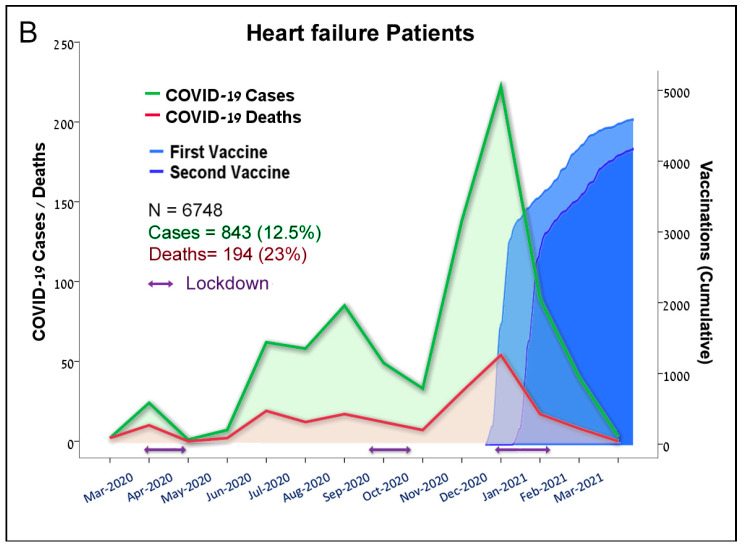
Timeline of 2020/2021 COVID-19 pandemic in Israel. (**A**) Three COVID-19 infection waves in the whole Israeli population as reported by the Israeli ministry of health and retrieved on 29 March 2021. Cases, deaths, and vaccinations are depicted. (**B**) Infection waves in the HF cohort. Data are depicted per month. Vaccination data are accumulative per day.

There were three waves of SARS-CoV-2 infection in Israel during the pandemic study period (Figure 1A). This paralleled with a corresponding peak infection rate in the HF patients (Figure 1B). These waves were associated with peaks of the death rates in the general and the HF cohort, which declined following three nationwide lockdowns. A nationwide vaccination campaign launched by the Israel Ministry of Health in December 2020 resulted in a dramatic decline in the incidence of SARS-CoV-2 infections thereafter (Figure 1A,B).

### 3.2. COVID-19 Pandemic in Heart Failure Patients

The study cohort included 6748 patients with a diagnosis of HF during the study period. During this period, 12.5% (843/6748) of the HF patients were infected with COVID-19, and 194 HF patients died from COVID-19, a 23% mortality rate. The clinical characteristics of the patients are presented in Table 1. The median age of the patients was 74 years old, and 55% of the patients were male. There was a higher percentage of patients with diabetes mellitus and obesity and fewer smokers in the group of HF patients infected with COVID-19 compared to the uninfected group. There was no difference in the percentage of patients with hypertension in the two groups (Table 1). Predictors of mortality in the HF patients infected with COVID-19 were increasing age, male sex, NYHA class, reduced renal function, and patients who did not receive renin–angiotensin system inhibitors (Table 2).

### 3.3. Clinical Management and Outcome during COVID-19 Pandemic

**Figure 2:**
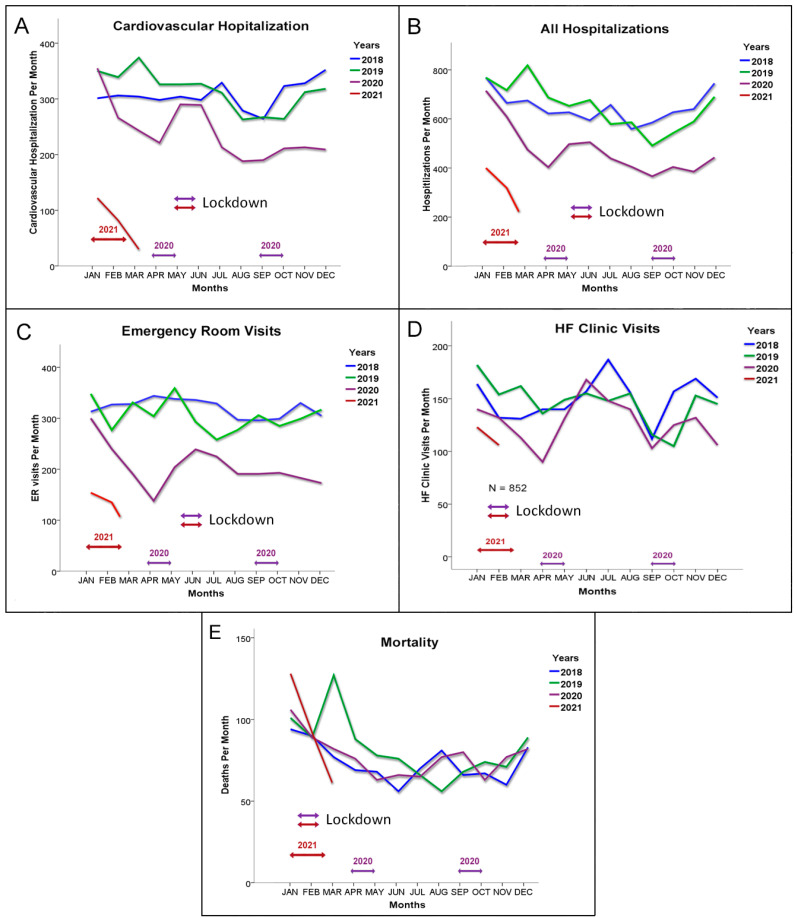
Health service usage during the pandemic compared to the two years prior to the pandemic. Data are presented as total numbers per month. (**A**) Cardiovascular hospitalizations. (**B**) All hospitalizations. (**C**) Emergency room visits. (**D**) HF clinic visits. Data are from the 852 patients followed at the Clalit health maintenance organization heart failure center in Jerusalem. (**E**) Mortality rates per month in the HF cohort.

**Figure 3:**
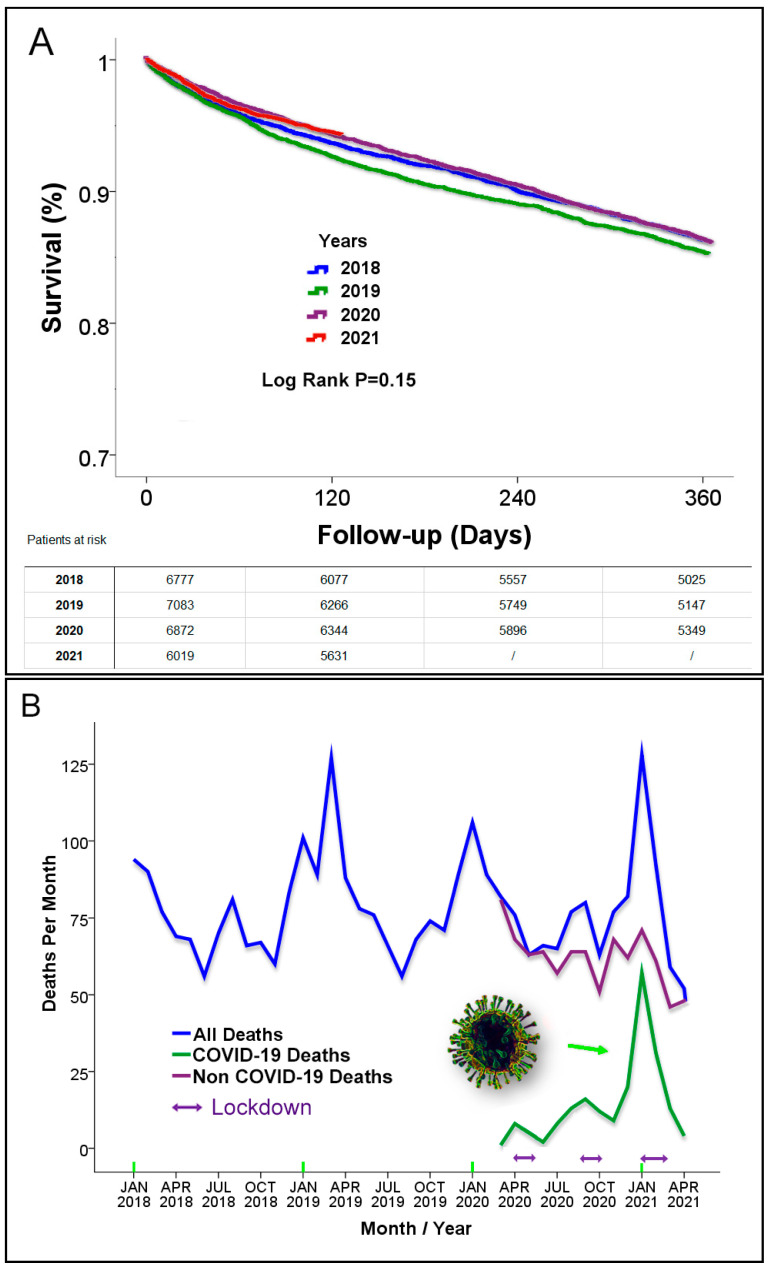
Survival data and death causes during and prior to the pandemic. (**A**) Kaplan–Meier survival curves for HF patients per year. Survival rates prior to the pandemic (2018–2019) and during the pandemic (2020). Survival rates were very similar: 86.2 ± 0.4% vs. 85.3 ± 0.4% vs. 86.0 ± 0.4%, respectively, *p* = 0.15. Survival data during the first quarter of 2021 are also depicted with a survival curve that was very similar to previous years but not included in the log-rank analysis. (**B**) Death rates per month prior to and during the pandemic. Deaths are divided by death due to COVID-19 and to other causes. A decline of the HF mortality deaths from other causes replaced with deaths due to COVID-19.

During the COVID-19 pandemic, there was a marked decrease in the utilization of medical services in the HF cohort. The number of cardiovascular hospitalizations as well as all hospitalization were significantly decreased compared to the two years prior to the pandemic (2018–2019), particularly during the lockdowns (Figure 2A,B). The number of emergency room visits was also significantly lower (Figure 2C). There was also an initial decrease in HF clinic visits per month, but this was partially restored over time (Figure 2D). This was due to introduction and application of remote visits after the first COVID-19 wave. Almost 40% of the visits were remote visits. Table 3 provides the statistical analysis that compared these clinical encounters between the pandemic period and previous years. Despite the reduced number of clinical encounters, the mortality rates of the HF patients were very similar during the pandemic compared to previous years (Figure 2E). In addition, the survival rate of the HF patients during follow-up in the study period was similar compared to previous years (*p* = 0.15; Figure 3A).

**Figure 4:**
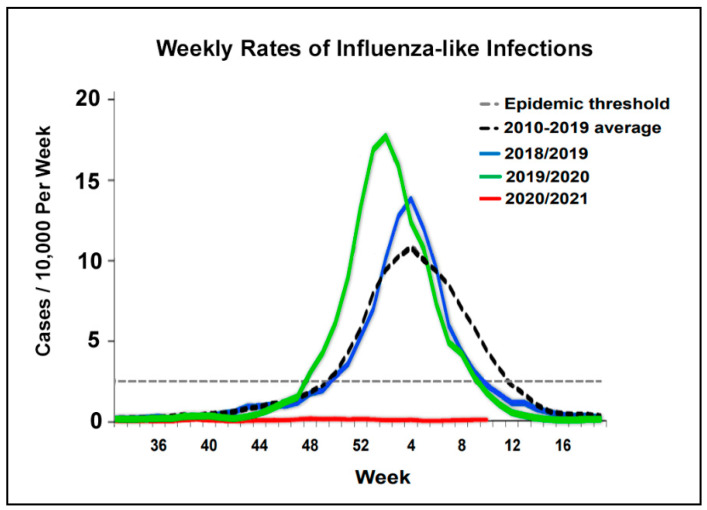
Weekly rates of influenza-like infections during the winter weeks. A noticeable absence of influenza-like infections during the pandemic of 2020/2021 compared to previous years and the previous ten-year average. Figure adapted from a published report of the Israel Center for Disease Control, Israel Ministry of Health [10].

An analysis of the death rates, separating death due to COVID-19 from death due to other causes, demonstrated a decline in HF mortality deaths from other causes, which was replaced with deaths due to COVID-19 (Figure 3B). A possible reason for the decline of non-COVID-19 deaths may be related to the reduced exposure to respiratory infections due to social distancing during the pandemic. A significant respiratory infection particularly during the winter season is the influenza virus. Data on the rate of influenza cases in Israel during the pandemic reported by the Israel Center for Disease Control, Israeli Ministry of Health [10] (Figure 4), demonstrated a remarkable drop of influenza cases during the COVID-19 pandemic period in Israel. Influenza cases were practically nonexistent during the pandemic.

## 4. Discussion

The present study looked at the clinical encounters as well as clinical outcomes in a cohort of HF patients in Jerusalem, Israel, during the COVID-19 pandemic of 2020–2021. The current study demonstrated that HF patients were somewhat more likely to be diagnosed with SARS-Cov2 viral infection compared to the general population in Israel (~10%) but that mortality rates were higher in HF patients; in this cohort, the mortality rate was 23%. In addition, there was a marked reduction in hospitalizations during the pandemic that included cardiovascular as well as total hospitalizations. Emergency ward visits were also reduced. HF clinic visits were initially reduced, but later on, they were replaced by remote visits. Despite the significant mortality in those patients infected with COVID-19, absolute mortality rates were not significantly different from previous years.

Several studies have previously reported a significant decline in HF hospitalizations during the COVID-19 pandemic [11,12,13,14,15], and one study reported that patients hospitalized during the pandemic had a higher mortality rate [12]. A recent publication [15] demonstrated a similar in-hospital mortality but an increased out of hospital mortality for HF patients during the pandemic. An analysis of the causes of death in that study demonstrated reduced deaths labeled as primarily occurring due to HF and increased deaths labeled as COVID-19 deaths as a principal cause of death [15].

The overall mortality rates in the present study in patients with HF were not significantly different from the mortality rates in the years prior to the pandemic. This finding was surprising, as the pandemic disrupted access to medical care and increased social isolation; as such, the pandemic would be expected to increase morbidity and mortality. This was not seen in the present study, at least not during the first year of the pandemic. This was also not seen in a Danish Nationwide Cohort Study [13] that demonstrated findings similar to those of the present study: reduced hospitalizations but similar mortality.

Why would this occur? While we do not have a definite explanation for the phenomena, there are several factors that may explain the findings. Death rates in patients who were not infected with COVID-19 were lower when compared to previous years, which suggests that changes during the pandemic and particularly during the lockdowns reduced hospitalization rates and non-COVID-19 deaths. Whereas some of the reduction in hospitalizations could be driven by postponement of treatment with negative clinical consequences, the data in the present study imply that at least part of the reduced hospitalizations was due to an actual reduction in morbidity in the HF patients during the pandemic. This may have been driven by reduced exposure to respiratory infections, lower levels of air pollution, and a reduction in other insults due to social distancing, staying at home, and the lockdowns. This is particularly evident with influenza viral infections, which were almost completely absent in Israel during the pandemic. However, caution is needed when interpreting the data, as it is possible that the reduced non-COVID-19 mortality was mainly due to events competing with COVID-19 infection.

The mortality rates of patients with HF that were infected with COVID-19 were very high, at 23%, nearly 10 times the mortality rate in the general population. This high mortality rate is very similar to data in HF patients published in the literature, with reported mortality rates from COVID-19 ranging from 24% [16] to ~40% [13,17]. The only available solution at this time to reduce this high mortality rate is prevention by readily available vaccinations. These vaccinations are highly effective and safe. The reduction in mortality seen after the initiation of vaccinations for COVID-19 suggests that the vaccinations were successful in preventing infection and subsequent mortality. This was definitively demonstrated in Israel [18], where the pandemic was drastically curbed by nationwide vaccinations.

Predictors of mortality from COVID-19 in the present study included increasing age and male sex. These have been previously reported as significant predictors of worse outcome in HF patients with COVID-19 [16,17] as well as important predictors in the general population infected with COVID-19. Other predictors such as NYHA class and reduced renal function have not been described before in the context of COVID-19 but are known predictors of reduced outcome in HF patients. Particularly interesting is the association between better outcome in patients treated with renin–angiotensin system inhibitors. These medications were hypothesized to increase susceptibility to SARS-CoV-2 infection at the beginning of the pandemic, as the therapy increases angiotensin-converting enzyme 2 expression in several tissues, and SARS-CoV-2 uses this receptor to gain entry into cells. There is no clinical evidence to support this. As these medications protect from lung injury in animal models, it has also been suggested that these medications may be beneficial in COVID-19 infection although data in the general population are not conclusive [19], and randomized studies are pending.

## 5. Limitations

Several potential limitations of this study merit consideration. The present study was an observational study. The study was from a community-based cohort in the Jerusalem district, which id insured by a health maintenance organization. The findings may not be applicable in other countries with different HF cohorts and in particular, different social and medical management systems. The present study did not have long-term clinical data beyond the first year of the pandemic and is possible that the pandemic may cause long-term cumulative detrimental clinical effects on HF outcomes that were not seen in the present study. Data regarding clinical parameters and drug therapy were extracted from a digitized database. Although this database has been validated and found to be highly accurate, not all data could be verified. While we tried to adjust for clinically relevant parameters, not all clinical parameters were available, and it is impossible to adjust for all variables that may affect outcome. Data on natriuretic peptide levels were not available.

## 6. Conclusions

The COVID-19 pandemic led to a significant reduction in medical services including hospitalizations and ER visits, particularly during lockdowns. High mortality rates were seen in HF patients infected with COVID-19, but overall mortality rates in HF patients did not increase. Further studies may help elucidate the factors that led to reduced mortality in non-infected HF patients. COVID-19 infection carries a very high mortality rate in HF patients, and all efforts should be sought to prevent infections in this very high-risk population.

## Figures and Tables

**Table 1:** Demographics and clinical characteristics of patients with heart failure according to COVID-19 infection.

Variable	No COVID-19 Infection (*n* = 5905)	COVID-19 Infection (*n* = 843)	Total (*n* = 6748)	*p* Value
Age (Years)	74 (64–83)	73 (63–82)	74 (64–83)	0.02
Gender (Male)	3239 (55)	448 (53)	3687 (55)	0.35
NYHA Class III/IV	1434 (33)	238 (37)	1672 (33)	0.06
HF Type				
Reduced ejection fraction	2406 (41)	391 (46)	2797 (41)	<0.001
Preserved ejection fraction	1935 (33)	274 (33)	2209 (33)	
Not-specified	1564 (26)	178 (21)	1742 (26)	
Hypertension	4785 (81)	697 (83)	5482 (81)	0.26
Diabetes mellitus	3098 (52)	508 (60)	3606 (53)	<0.001
Obesity	3091 (52)	501 (59)	3592 (53)	<0.001
Hyperlipidemia	5242 (89)	745 (88)	5987 (89)	0.71
Ischemic Heart Disease	3813 (65)	534 (63)	4347 (64)	0.48
Prior Myocardial Infarction	2359 (40)	312 (37)	2671 (40)	0.1
Peripheral vascular disease	783 (13)	100 (12)	883 (13)	0.13
Prior Stroke/transient ischemic attack	1279 (22)	202 (24)	1481 (22)	0.13
Chronic obstructive lung disease	1134 (19)	150 (18)	1284 (19)	0.33
Chronic Renal Failure	1575 (27)	280 (33)	1855 (27)	<0.001
Dialysis	269 (5)	84 (10)	353 (5)	<0.001
Dementia	717 (12)	96 (11)	813 (12)	0.53
Depression	950 (16)	132 (16)	1082 (16)	0.26
Malignancy	1311 (22)	164 (19)	1475 (22)	0.07
Smoker (past or current)	2931 (50)	351 (42)	3282 (49)	<0.001
Current Smoker	2234 (38)	261 (31)	2495 (37)	<0.001
Body mass index (kg/m^2^)	29 (25–33)	30 (26–35)	29 (25–33)	<0.001
Systolic blood pressure (mmHg)	129 (120–140)	129 (120–140)	129 (120–140)	0.98
Diastolic blood pressure (mmHg)	72 (65–80)	73 (67–80)	72 (65–80)	0.37
Pulse (beats per minute)	72 (65–80)	73 (65–80)	72 (65–80)	0.63
Laboratory Data				
Urea (mg/dL)	42 (32–58)	44 (34–65)	42 (33–59)	0.002
Creatinine (mg/dL)	0.9 (0.8–1.2)	1.0 (0.8–1.4)	0.9 (0.8–1.3)	0.01
Estimated glomerular filtration rate (mL/min per 1.73m^2^) *	74 (53–95)	71 (50–93)	73 (53–94)	0.01
Medication				
RAS inhibitors	4763 (81)	656 (78)	5419 (80)	0.05
Beta blockers	4629 (78)	668 (79)	5297 (78)	0.57
Spironolactone	2415 (41)	343 (41)	2758 (41)	0.91
Furosemide	3917 (66)	602 (71)	4519 (67)	0.003
Thiazide	1017 (17)	130 (15)	1147 (17)	0.19
Digoxin	373 (6)	58 (7)	431 (6)	0.53
Amiodarone	992 (17)	137 (16)	1129 (17)	0.69
Aspirin	3585 (61)	528 (63)	4113 (61)	0.28

Data are presented as median (interquartile range) for continuous variables and counts (percentages) for categorical variables. *p* value determined by the Kruskal–Wallis test for continuous variables and the Chi-square Test for categorical variables. Diabetes mellitus defined as fasting plasma glucose ≥126 mg/dL or glucose lowering treatment; hypertension defined as blood pressure >140/90 mmHg measured on several occasions or anti-hypertensive treatment; and hyperlipidemia defined as low-density lipoprotein >130 mg/dL, fasting serum triglycerides >200 mg/dL, or lipid lowering treatment. * Estimated glomerular filtration rate was calculated using the modified modification of diet in renal disease (MDRD) equation (175 * serum creatinine^−1.154^ * age^−0.203^. For females, a correction factor is used, multiplying by 0.742.). RAS inhibitors include renin–angiotensin system inhibition by angiotensin converting enzyme inhibitors, angiotensin receptor blockers, or angiotensin receptor–neprilysin inhibitors. HF, heart failure; NYHA, New York Heart Association.

**Table 2:** Predictors of mortality by Cox regression analysis.

	Univariable		Multivariable	
	Hazard Ratio (95% CI)	*p* Value	Hazard Ratio (95% CI)	*p* Value
Age (years)	1.06 (1.04–1.07)	<0.001	1.05 (1.04–1.07)	<0.001
Gender (Male)	1.11 (0.84–1.46)	0.47	1.46 (1.09–1.96)	0.01
NYHA III/IV	1.60 (1.18–2.17)	0.003	1.53 (1.11–2.12)	0.01
Diabetes Mellitus	1.32 (0.99–1.76)	0.06	1.25 (0.91–1.71)	0.17
Hypertension	2.09 (1.32–3.32)	0.002	1.13 (0.69–1.85)	0.64
Ischemic Heart Disease	1.41 (1.05–1.89)	0.02	1.09 (0.80–1.50)	0.58
BMI *	0.22 (0.05–0.92)	0.04	0.31 (0.05–1.70)	0.18
eGFR ** (mL/min per 1.73 m^2^)	0.32 (0.23–0.45)	<0.001	0.38 (0.26–0.56)	<0.001
RAS Inhibition	0.63 (0.47–0.85)	0.003	0.68 (0.49–0.95)	0.02

Data are presented as hazard ratio (95% confidence interval), *p* value. * Log-transformed; ** Square root-transformed; Parameters that were included in the multivariable analysis model were age, gender, NYHA class, diabetes, hypertension, ischemic heart disease, log-transformed body mass index, square root-transformed estimated glomerular filtration rate and RAS inhibition.

**Table 3:** Medical service usage during the pandemic (2020–2021) and previous years in the HF cohort.

	2018 (*n* = 365)	2019 (*n* = 365)	2020 (*n* = 366)	2021 (*n* = 89)	Total (*n* = 1185)	*p* Value
CV hospitalizations per day	10 (7.0–12)	10 (8.0–13)	7.0 (5.0–10)	3.0 (2.0–4.0)	9.0 (6.0–12)	<0.001
Length of stay (Days)	5.0 (4.0–6.0)	5.0 (4.0–6.0)	5.0 (3.0–6.3)	6.0 (3.0–8.0)	5.0 (3.5–6.0)	0.35
Hospitalizations per day	21 (17–25)	21 (17–26)	15 (12–19)	10 (6.0–14)	18 (14–23)	<0.001
ER visits per day	10 (8.0–13)	10 (7.0–12)	7.0 (4.0–9.0)	4.0 (2.0–6.0)	9.0 (6.0–11)	<0.001
HF Clinic Visits per working day *	6.0 (4.0–10)	6.0 (4.0–10)	5.0 (0.0–10)	6.0 (3.0–8.0)	6.0 (3.0–10)	0.05
Deaths per day	3.0 (2.0–4.0)	3.0 (2.0–4.0)	3.0 (2.0–4.0)	3.0 (2.0–4.0)	3.0 (2.0–4.0)	0.60

Data presented as median (Interquartile range); *p*-value calculated by the Kruskal–Wallis nonparametric test. *n* refers to number of days during the year; * Patients that were followed in the HF clinic during the 3.25 years (*n* = 852). CV, cardiovascular; ER, emergency room.

## Data Availability

The data of this study are available from Clalit Health Service but restrictions apply to the availability of these data, which were used under the license for the current study, and are not publicly available. Data requests can be addressed to: The Institutional Ethics Committee for Human Studies of Clalit Health Services (email: meirhelsinki@clalit.org.il). Meir Medical Center. Tchernichovsky St 59, Kfar Saba, 4428164, Israel.

## Abbreviations

BMI	body mass index
CI	confidence intervals
CV	cardiovascular
eGFR	estimated glomerular filtration rate
ER	emergency room
HF	heart failure
HMO	health maintenance organization
NYHA	New York Heart Association
RAS	renin–angiotensin system
